# Supplementary material for: Prediction of Subsequent Contralateral Patellar Dislocation after First-Time Dislocation Based on Patellofemoral Morphologies
Source: J Clin Med. 2022 Dec 26;12(1):180. doi: 10.3390/jcm12010180 (PMC9820933; doi:10.3390/jcm12010180)
Supplement: Supplementary file 1 [file jcm-12-00180-s001.zip › Figure S2.pdf]

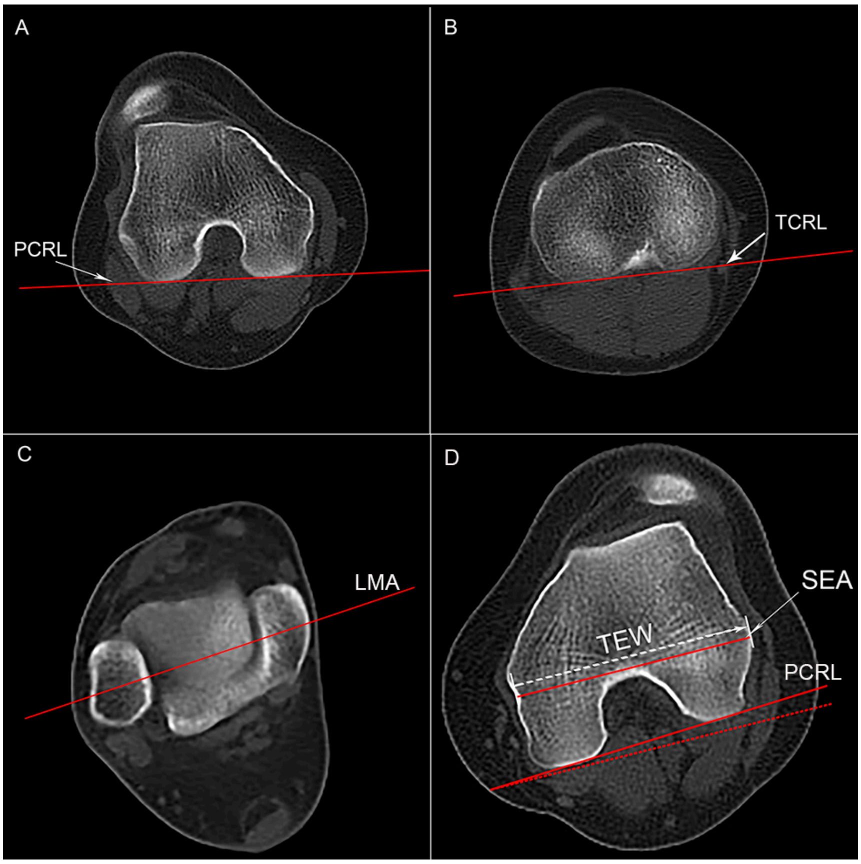

Figure S2. (A) The posterior femoral condylar reference line (PCRL) is shown. (B) The most proximal axial slice with intact bilateral tibial condyles, showing the line tangent to the condyles (TCRL). (C) A line is drawn through the center of the medial and lateral malleolus (LMA) on the slice which showed the medial and lateral malleolus clearly. Superimposing A-B or B-C to calculate knee joint rotation (the angle between PCRL and TCRL) and tibial torsion (the angle between TCRL and LMA). (D) PCRL, the line through the sulcus of the medial epicondyle and the prominence of the lateral epicondyle (SEA), and the red dotted line parallel to SEA are shown. The width of SEA (TEW). Posterior condylar angle (PCA) is the external rotation angle of SEA relative to PCRL.
