# Supplementary material for: Prediction of Subsequent Contralateral Patellar Dislocation after First-Time Dislocation Based on Patellofemoral Morphologies
Source: J Clin Med. 2022 Dec 26;12(1):180. doi: 10.3390/jcm12010180 (PMC9820933; doi:10.3390/jcm12010180)
Supplement: Supplementary file 1 [file jcm-12-00180-s001.zip › Figure S3.pdf]

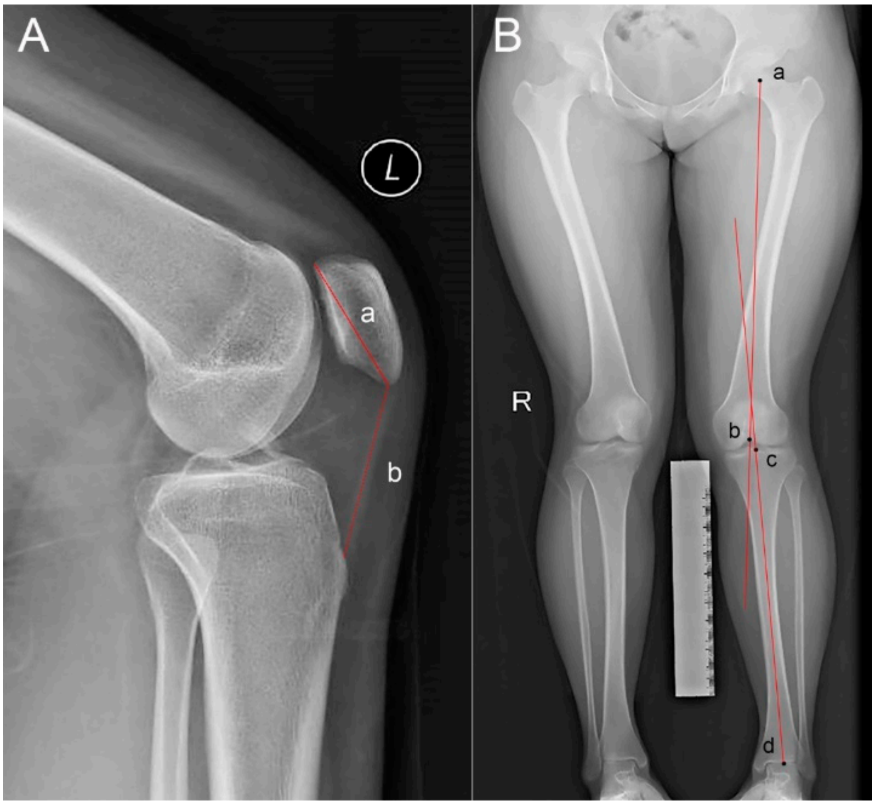

Figure S3. (A) Insall-Salvati index (ISI) is defined as the ratio of the patellar tendon (b) to maximum length measured from the distal pole to the proximal pole of the patella (a). (B) Hip-knee-ankle (HKA) angle measured by weight-bearing full-leg radiographs. The points a, b, c, and d represent to the center of the femoral head, femoral condyles, tibial plateau, and ankle joint, respectively. It is defined as the angle between the mechanical axis of the femur (a-b) and the mechanical axis of the tibia (c-d).
