# Supplementary figures and images for: Prediction of Subsequent Contralateral Patellar Dislocation after First-Time Dislocation Based on Patellofemoral Morphologies
Source: J Clin Med. 2022 Dec 26;12(1):180. doi: 10.3390/jcm12010180 (PMC9820933; doi:10.3390/jcm12010180)

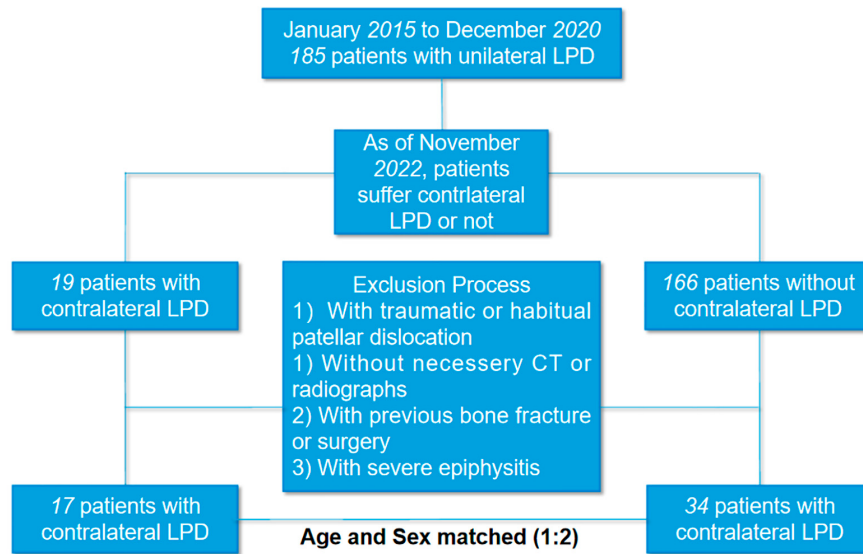

**Figure S1.** Flowchart.

Supplement: Supplementary file 1 [file jcm-12-00180-s001.zip › Figure S1.pdf]
